# Supplementary material for: Belonging through meaningful activity in the transition from unhoused to housed
Source: PLoS One. 2024 Sep 26;19(9):e0310701. doi: 10.1371/journal.pone.0310701 (PMC11426477; doi:10.1371/journal.pone.0310701)
Supplement: Attachment — Submitted filename: Response to reviewers 08 30 2024.docx [file pone.0310701.s002.docx]

Dear Dr. Katie Gibson Smith,

Thank you for considering our paper on belonging through meaningful activity in the transition from unhouse to housed for PLOS One. Please see the Response to Reviewers below.

Regarding data availability, I am including a Supporting Information File that includes the coding tree. In this way, it is possible to maintain confidentiality and any reader who would like to look at the data analysis may do so.

[Double check formatting / file naming is correct]

| Belonging through meaningful activity in the transition from unhoused to housed – Response to Reviewers | |
| --- | --- |
| Reviewers’ Suggestion | Response |
| Reviewer #1 |  |
| Manuscript generally written well. My comments are mainly minor. | We are pleased that the reviewer found that the manuscript was generally well-written and that the comments are mainly minor. |
| Abstract: |  |
| Meaningful activity not defined. | Added the following to the background section of the abstract: Meaningful activities are things we do that bring value to our lives. |
| Aim: please use SPIDER framework to write aim for qualitative studies. Also in the main text. | In the abstract, we revised the Purpose statement as follows:  To explore how engaging in meaningful activities may ~~facilitate~~ influence experiences of belonging following homelessness through a secondary analysis of qualitative interviews.  In the main text, the research question statement was revised as follows:  To address this gap in existing literature, we conducted ~~this research~~ a qualitative thematic analysis guided by the question: “How does engaging in meaningful activities influence experiences of belonging for individuals transitioning from unhoused to housed?”  Note that the first section in Methods section states that we conducted a secondary qualitative data analysis. To avoid repetition, I used ‘qualitative thematic analysis’ to indicate the ‘D’ and ‘R’ for the research statement in SPIDER format. |
| Method: provide further details about the design, participant identification and recruitment, sampling etc. | Revised Methods section in the Abstract:  Using interviews conducted in a community-based participatory action study exploring the transition to housing following homelessness (n=19), we conducted a thematic analysis using the method described by Braun and Clarke. Participants were recruited through communication with local organizations supporting individuals with lived and living experiences of being unhoused as well as through presentations at drop-in organizations. An intentional effort was made to recruit diverse participants regarding housing status, age, and gender. Inductive analysis was use to conduct initial coding, focusing on belonging and engaging in meaningful activities. We then analyzed the codes abductively, ~~used~~ using Bourdieu’s Social Capital Theory to inform this analysis. |
| Results: perhaps provide theme headings as it is difficult for readers to make sense of key results from the ‘essence’ presented. | We added theme headings to the abstract:  Within this overall essence, we generated three themes: 1) Human connection: “being where I am with people who care about me, I actually feel good”; 2) Social exclusion: being a “regular member of society”; and 3) Non-human connection: “my cats…are like my kids to me.” |
| Main text: |  |
| Define meaningful activities here. | We added the following section to the main text:  **What is meaningful activity and how does it relate to health and well-being?**  Meaningful activities are things we do that bring value to our lives, where the meaning is subjective for each person [18-20]. An applicable definition is: “all the everyday things we do in our life roles, but also the things we do to create a meaningful life and to engage with wider society and culture” [21]. Several aspects denote the concept of ‘meaningful,’ such as experiencing joy and pleasure, a sense of purpose, and meeting personal needs where individuals can experience choice, control, and belonging [18, 22]. Meaningful activities can bring a sense of connection to oneself, to others, and to the environment [19] and can positively impact mental, physical, and psychosocial well-being and life satisfaction [23]. However, when individuals lack opportunities to engage in meaningful activities, they can experience boredom, depressive symptoms, and diminished physical well-being [23, 24]. Of particular significance for individuals transitioning out of homelessness is that there is a high risk of experiencing boredom when individuals are unhoused, which may increase poor mental health, substance use, and hopelessness [24]. |
| In the background mention resource related barriers to engaging in meaningful activities plus the importance of comorbidities. | We added a paragraph following the above-noted paragraph on meaningful activities:  Individuals exiting homelessness often experience barriers to engaging in meaningful activities such as living in low-income, a lack of access to transportation, and ongoing social exclusion [6, 7, 25, 26]. Living in low-income limits opportunities to engage in activities in the community, including accessing public transportation, unless there are free or low-cost options available [7, 27]. Those living with substance use disorder, mental illness, and physical disabilities face additional challenges to engaging in meaningful activities. For example, individuals experiencing mental illness symptoms may struggle to reconnect with friends and family [28] and those in substance use recovery may be rebuilding their social support network while avoiding going to places where they may be triggered to use substances [29]. Going out into the community to engage in activities is difficult for some individuals living with mobility impairments due to getting exhausted quickly, experiencing difficulty getting around the community, or not being able to get out of the house [30]. |
| In the methods, please describe study settings, participant recruitment and consent processes. Referring to the parent study is not enough for the readers. | Additional information was added regarding the settings as well as the recruitment process is included in the revised paragraph below:  The parent study received ethics approval from both Western and Queen’s Universities in Ontario, Canada. Participants were purposively recruited from shelters, permanent and transitional supportive housing programs, and drop-in centres that supported individuals with homelessness experiences in two mid-sized Canadian cities, Kingston and London, ON~~, Canada~~. Recruitment from these settings included: 1) sending emails to the leaders of the health and social care organizations with details about the study and requesting support with the recruitment process; 2) providing presentations to the staff of relevant organizations, encouraging them to provide contact information regarding this study to potential participants; and 3) attending drop-in sessions at local organizations where individuals could ask questions and learn more about the study. An intentional effort was made to recruit diverse participants regarding housing status, age, and gender.  The consent process description was revised as follows:  Interviewers read a letter of information about the study and the request for consent out loud and verbally sought consent prior to conducting interviews.  The reasoning behind why informed consent was conducted verbally is stated later in the paragraph:  All interview components were read aloud to all participants to avoid threats to trustworthiness related to low literacy. [no changes were made to this sentence; including for information only] |
| Explain the high reimbursement rates. | Added the following:  Participants received $40 for participating in the interviews, acknowledging the value of their time committed to this contribution. In participatory action research, it is important to ensure all participants know that they are valued and contributing members of the team. Therefore, participants were compensated for their time at a comparable amount to paid research team members. |
| What if participant chosen pseudonyms would identify them? I have not seen this practice before. | In the Semi-structured qualitative interviews section, added:  While unconventional, inviting participants to choose their pseudonyms provides a way to experience ownership and a sense of contribution to the study and offers the opportunity to include part of themselves while maintaining a balance of autonomy and confidentiality [38]. |
| Suggest avoid referring participants with ‘his’ ‘her’, use neutral tone (e.g. their) to further ensure anonymity. | Revised the Qualitative Findings section to use a neutral tone (throughout the section) |
| On the theme ‘being a regular member of the society’- please describe if there was any roles or scope for healthcare professionals that participants identified which would facilitate this process. Perhaps this reference can help https://doi.org/10.3399/bjgp18X694577 to discuss this aspect. | Within theme 2, we added:  In particular, Barbara found the continued support she received by attending their substance use recovery program as a stabilizing factor that allowed them to work.  Several participants were either studying or wanting to work in a field such as assisting with navigating the legal system, supporting individuals through their substance use recovery journey, and providing counselling support, where they could give back to the community. They found these supports helpful in their journey and wanted to be able to help others in similar situations. |
| In the discussion, please incorporate some literature on how arts based interventions e.g. arts, gardening could be meaningful activities to engage people who have experienced homelessness- cite some references and learnings available. As this aspect seem to be missing. | Added the following paragraph:  Strategies for engaging with the living world include gardening and various arts-based activities such as using materials from nature to create are or painting scenery. Community gardening has the potential to meet several needs for individuals as they transition from homelessness. Gardens provide a source of low-cost and healthy food as well as opportunities to build connection and community and a sense of ownership [37]. Hamilton et al. [37] developed a community-based program for individuals who transitioned out of homelessness that included groups where individuals built social connections, received resources such as a community-made cookbook, had access to kitchenware, and grew their own garden produce. Engaging in creative arts has been shown to be beneficial for individuals with living experience of homelessness, providing opportunities to connect with their culture, as a way to connect with oneself, and as a way to share experiences and explore spirituality [38]. During homelessness, the lack of housing was a barrier [38]; however, it is possible that individuals who are recently housed may experience similar benefits and have a space they can engage with these activities on their own as well as with others as way to experience belonging. |
| Reviewer #2 |  |
| Overall, this has been written to a high standard and is clear and well written throughout. The manuscript engages with an important topic and explores the concept of ‘belonging’ in regards to individuals with experiences of being unhoused. Important and insightful work from those with lived and living experience is presented and the authors have generated rich insight into how ‘meaningful activities’ develop a sense of belonging. | We appreciate the overall positive feedback for this manuscript and we are glad that this work conveys the importance of exploring belonging among individuals with lived and living experience of being unhoused and the link to meaningful activities in developing a sense of belonging. |
| The context and rationale are well-described and the aims of the research are clear with a solid methodology. I have some minor points in regards to the presentation of the themes within the findings and areas for improvement (see below points). The discussion and conclusion provide a robust summary of the research. | We value this positive feedback and the suggestions outlined below to improve this paper. |
| Introduction: |  |
| 1. While you have explored the context of ‘belonging’ within the introduction, linking this to health and well-being, the ability to integrate into a community and Bourdieu’s Social capital Theory, it would be helpful to provide more clarity in regards to ‘meaningful activities’ as this is a key aspect of your research objective. Meaningful activities are briefly explored in (lines 101-107), but more detail would enable the reader to gain a sense of your interpretation and definition of what this aspect means within the context of your study. | See above comment regarding adding a section focused on meaningful activities. |
| Methods: |  |
| 2. Recruitment (line 138). It would be valuable to acknowledge any potential sampling bias due to purposive recruitment and what steps were taken to mitigate against this. Addressing this will enhance the credibility and rigour of the study design. | Added the following to the Recruitment section:  Possible biases in recruitment include missing individuals who transitioned to independent housing without the support of health and social care organizations, missing individuals who were experiencing hidden homelessness such as couch surfing prior to becoming housed, and an overrepresentation of individuals who seek out support. Also, the recruitment approach of staff at the various organizations inviting potential participants added a layer of possible bias as the staff were making the decisions regarding who would be invited to participate. To mitigate the possible biases, several approaches to recruitment, as noted above, were incorporated to inform the widest range of participants about the study. |
| 3. Analysis (line 162). The study described using thematic analysis to analyse interview transcripts, drawing from Braun and Clarke (2006). Given that TA is utilised extensively across qualitative research, it would be helpful to provide more detail to explain how this approach was applied within the analysis of this specific study. There are now many different versions of TA available to qualitative researchers since the 2006 publication and acknowledging that would provide further detail to justify your particular TA approach. It would also be useful to provide further clarity in regards to the process used, for example, was this an inductive or deductive approach? | We provided further information regarding the TA process:  In the secondary analysis presented in this paper, we used thematic analysis to analyze interview transcripts [26, 27]. We used inductive analysis to conduct the initial coding and themes. We conducted three rounds of coding, focusing on belonging and engaging in meaningful activities, then grouped codes into like categories [26]. The categories were then arranged into themes and refined through discussions. Finally, we identified an essence and further refined our analysis. We used Dedoose, a cloud-based data management program, to organize the data [27] and finally, to abductively code transcripts informed by SCT [19]. We also created a visual representation of the data to help us to better understand the data (see Fig 1). |
| Qualitative Findings: |  |
| 4. Presentation of themes throughout the manuscript (Abstract and Findings). While I found the quotes powerful to convey the themes generated, I would have liked an inclusion of a name or definition alongside the thematic quotes. This would provide greater clarity in formulating your interpretation of each theme to convey explicit understanding of the data. For example, is Theme 1 speaking to the importance of ‘connection’ or ‘relationships’? Is Theme 2 speaking to ‘social exclusion’ or ‘discrimination’? Is Theme 3 speaking to the importance of non-human interactions? The quote for theme 3 highlights the importance of pets, but also discusses nature as an important aspect, but this is not captured in the overall essence of the theme. Without some contextualisation of the themes by defining and naming them, alongside the quotes, the reader is having to interpret this themselves. | We added a short name for each theme (added to Abstract and Findings):  Theme 1~~:~~ . Human connection: “Being where I am with people who care about me, I actually feel good” [Michelle].  Theme 2~~:~~ . Social exclusion: Being a “regular member of society” [Barbara].  Theme 3~~:~~ . Non-human connection: “My cats…are like my kids to me” [Gavin]. |
| Discussion: |  |
| 5. Line 524. This sentence seems to end abruptly when highlighting that multiple contextual factors limited opportunities to belong. It would be helpful to provide more detail in regards to this, what are the contextual factors, a brief summary here for clarity for the reader. | We expanded this sentence to provide examples of contextual factors:  However, multiple contextual factors limited opportunities to belong, such as living in low-income, challenges with reconnecting with family members and friends, and health and disability-related factors that made it difficult for participants to engage in activities within the community where they would have more opportunities to meet people. |
| Practice Implications: |  |
| 6. Line 584. More detail in regards to what is meant by ‘service providers’ as this is unclear. Do you mean health and social care services? Community organisations? This will enable you to connect to the context and be helpful for the international audience. | Rephrased this to read:  Health and social care providers supporting individuals transitioning … |
| Limitations: |  |
| 7. Line 616-621. It was good to see you acknowledge the limitations within this study, such as the majority of participants identifying as White. However, it would be helpful to provide further rationale in regards to why this was the case in your study and what steps, if any were taken to ensure a diverse sample. What can be done to address this gap within future research? | Added the following information to the Limitations section:  However, it is important to consider why representation from non-White groups, and specifically from Indigenous persons because Indigenous persons make up such a high proportion of unhoused individuals in Canada. Typical approaches such as Housing First have not supported Indigenous peoples well [49]. It is possible that, for similar reasons, potential Indigenous participants may not have been involved with the community organizations that were approached for recruitment purposes. However, Indigenous researchers [47] recommend collaborations between organizations supporting Indigenous persons and academic researchers to ensure the needs of Indigenous peoples are met and that there is funding available to conduct the research. Additionally, studies focused on Indigenous peoples should include Indigenous persons as part of the research team [50]. |
| Changes to reference list | Added references for the new Meaningful Activity section: #18-30  We noticed an incorrect reference in the Reference list: Replaced the incorrect Marshall et al. (2022) with the correct Marshall et al. (2023) – Reference number: 36  Added the following references:  In the Analysis section: #40  In Discussion section: #50-51  In Limitations: #62-63 |
